# RSH enzyme diversity for (p)ppGpp metabolism in *Phaeodactylum tricornutum* and other diatoms

**DOI:** 10.1038/s41598-019-54207-w

**Published:** 2019-11-27

**Authors:** Luisana Avilan, Carine Puppo, Adrien Villain, Emanuelle Bouveret, Benoit Menand, Ben Field, Brigitte Gontero

**Affiliations:** 1 Aix Marseille Univ CNRS, BIP, UMR 7281, IMM FR 3479, 31 Chemin Joseph Aiguier, 13009 Marseille, France; 2 Aix Marseille Univ CNRS, IGS; UMR 7256, IMM FR 3479, 13009 Marseille, France; 3 Stress Adaptation and Metabolism in Enterobacteriae group, Department of Microbiology, Institut Pasteur, Paris, 75015 France; 4 Aix Marseille Univ, CEA, CNRS, UMR7265 BIAM, 13009 Marseille, France

**Keywords:** Phylogenetics, Plant evolution

## Abstract

The nucleotides guanosine tetraphosphate and pentaphosphate (together known as (p)ppGpp or magic spot) are produced in plant plastids from GDP/GTP and ATP by RelA-SpoT homologue (RSH) enzymes. In the model plant Arabidopsis (p)ppGpp regulates chloroplast transcription and translation to affect growth, and is also implicated in acclimation to stress. However, little is known about (p)ppGpp metabolism or its evolution in other photosynthetic eukaryotes. Here we studied (p)ppGpp metabolism in the marine diatom *Phaeodactylum tricornutum*. We identified three expressed *RSH* genes in the *P. tricornutum* genome, and determined the enzymatic activity of the corresponding enzymes by heterologous expression in bacteria. We showed that two *P. tricornutum* RSH are (p)ppGpp synthetases, despite substitution of a residue within the active site believed critical for activity, and that the third RSH is a bifunctional (p)ppGpp synthetase and hydrolase, the first of its kind demonstrated in a photosynthetic eukaryote. A broad phylogenetic analysis then showed that diatom RSH belong to novel algal RSH clades. Together our work significantly expands the horizons of (p)ppGpp signalling in the photosynthetic eukaryotes by demonstrating an unexpected functional, structural and evolutionary diversity in RSH enzymes from organisms with plastids derived from red algae.

## Introduction

Plastids, the defining feature of photosynthetic eukaryotes, arose from the endosymbiosis of a cyanobacterium more than one billion years ago^1^. Massive cyanobacterial gene loss occurred following endosymbiosis, along with the transfer of genes to the nuclear genome of the eukaryotic host. Plastids now retain a small genome that encodes housekeeping and photosynthetic proteins, as well as a bacteria-like gene expression machinery. Bacteria-like regulatory systems that may be involved in acclimation to environmental perturbation are also present in plastids^2,3^. One of these regulatory-systems is mediated by the nucleotides guanosine tetraphosphate and pentaphosphate (referred to as (p)ppGpp hereafter) whose levels are controlled by the antagonistic action of RelA-SpoT homologues (RSH). In bacteria, (p)ppGpp was originally identified as a magic spot on thin-layer chromatography plates in the 1960s^4^. Now bacterial (p)ppGpp signalling is well characterised: (p)ppGpp accumulates in response to stress to reduce proliferation and activates acclimatory pathways by targeting enzymes involved in transcription, translation, and replication^5,6^.

In the photosynthetic eukaryotes, (p)ppGpp was discovered more recently, and (p)ppGpp signalling has principally been studied in flowering plants. Although (p)ppGpp accumulates in response to various different stresses^7,8^, the actual role of (p)ppGpp during stress acclimation is not yet clear. However, (p)ppGpp is known to act as a potent inhibitor of plastid gene expression *in vivo*^9–11^, and altering the capacity of a plant to make (p)ppGpp influences photosynthetic capacity, growth and development^10,11^. Notably, (p)ppGpp appears to be important for regulating the equilibrium between the plastidic and nucleocytoplasmic compartments of the plant cell. Phylogenetic studies support the existence of at least three conserved families of plastid-targeted RSH enzymes in land plants named RSH1, RSH2/3 and RSH4^12^. The model flowering plant *Arabidopsis thaliana*, where plant (p)ppGpp homeostasis is the most well understood, possesses representatives from each of these families: RSH1 that lacks (p)ppGpp synthetase activity and appears to function as the major (p)ppGpp hydrolase^11^, the closely related RSH2 and RSH3 that appear to act as the major (p)ppGpp synthetases^10,11,13^, and a calcium- activated RSH (CRSH) from the RSH4 family that possesses a C-terminal EF-hand domain implicated in calcium binding, and has calcium-dependent (p)ppGpp synthesis activity *in vitro*^14^.

Plastids are the result of primary endosymbiosis, where a photosynthetic bacterium was engulfed by a eukaryote. Today several primary plastid lineages can be identified which are thought to share common ancestry. Plants and green algae (together known as the Viridiplantae) possess primary plastids of the green lineage. The Viridiplantae have two sister groups with primary plastids, the red algae (Rhodophyta) and glaucophytes (Glaucophyta). Primary plastids from the green and red lineages have also been transferred and mixed in new eukaryotic hosts to result in the astonishing range of plastid diversity that can be observed in nature^15^. Plastid-targeted RSH enzymes from the RSH1, RSH2/3 and RSH4 families have been identified in many green and red algae, yet the metabolism and functions of (p)ppGpp in these organisms have barely been investigated^3,12,16^. One exception is a recent report on the red alga *Cyanidioschyzon merolae*, where CmRSH4b, a member of the RSH4 family, was shown to possess (p)ppGpp synthetase activity^17^. Interestingly, the inducible expression of CmRSH4b in *C. merolae* results in a reduction in plastid size and rRNA transcription in a similar manner to the expression of a (p)ppGpp synthetase in Arabidopsis^11^.

Diatoms (Bacillariophyceae) are a group of golden brown coloured microalgae that contain complex plastids that originate from the secondary endosymbiosis of a red alga, with the addition of nuclear-encoded plastid-targeted green algal proteins left over from a previous endosymbiosis^18,19^. Diatoms are the predominant photosynthetic eukaryote in the oceans, where they account for around 40% of net primary production^20^. Therefore, understanding (p)ppGpp synthesis in diatoms, where it is likely to regulate photosynthetic capacity^10,11^, and play roles in diatom lifestyle, is of particular importance. To tackle this issue, we investigate (p)ppGpp metabolism in the model pennate marine diatom *Phaeodactylum tricornutum*. We first identified expressed *RSH* genes in the *P. tricornutum* genome and then determined if their gene products have (p)ppGpp synthetase/hydrolase activity by complementation of *Escherichia coli* (p)ppGpp biosynthesis mutants. Then, we place the structural and catalytic features of *P. tricornutum* RSH enzymes into an evolutionary context. Altogether our study advances our previously poor understanding of (p)ppGpp metabolism in diatoms.

## Results

### The nuclear genome of the model diatom Phaeodactlyum tricornutum encodes three RSH enzymes

**Figure 1:**
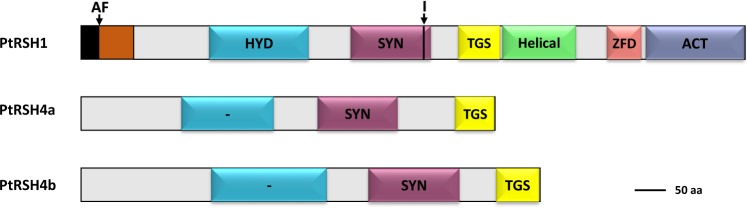
Primary structure of RSH from *P. tricornutum*. Schematic representation of domains found in *P. tricornutum* PtRSH1, PtRSH4a and PtRSH4b (JGI gene accession numbers 11099, 7629 and 33947): (p)ppGpp hydrolase (HYD), (p)ppGpp synthetase (SYN), Threonyl-tRNA synthetase GTPase Spot (TGS), zinc finger domain (ZFD) and Aspartate kinase, Chorismate mutase, TyrA (ACT). The bipartite peptide for targeting to the chloroplast is shown. Arrows indicate the cleavage site (AF) of the putative chloroplast signal peptide and the intron position (I) on the corresponding gene.

We inspected the *P. tricornutum* genome for the presence of *RSH* genes using BLAST and identified three: *PtRSH1*, *PtRSH4a* and *PtRSH4b*. *PtRSH1* is the only *RSH* gene that contains an intron. We analysed the predicted protein sequences derived from the three genes and identified a number of conserved domains that are typical of RSH enzymes (Figs. 1A, S1). PtRSH1 possesses several potential translation start sites that are supported by ESTs. Using the first translation start site we identified a bipartite signal peptide using ASAFind^21^. Although we identified N-terminal extensions before the first catalytic domain (Figs. 1A, S2), PtRSH4a and PtRSH4a did not possess bipartite signal peptides that could be identified by ASAFind. However, LOCALIZER, an algorithm not designed for identifying diatom bipartite target peptides though capable of detecting internal chloroplast targeting peptides^22^, predicted the presence of a chloroplast target peptide in PtRSH4a. There is also a body of indirect evidence that suggests the localisation of RSH enzymes within the chloroplast including the presence of RSH only within the photosynthetic eukaryotes^3^, and the experimental demonstration of chloroplast targeting for RSH from plants^10,14^ and red algae^17^. However, without more direct evidence we cannot conclude that all RSH from *P. tricornutum* are chloroplast targeted.

PtRSH4a and PtRSH4b bear (p)ppGpp hydrolase and synthetase domains that show signs of catalytic inactivation (Fig. S2). The (p)ppGpp hydrolase domains are divergent compared to the hydrolase domains of known (p)ppGpp hydrolases, and lack many residues critical for hydrolase activity^23^. While PtRSH4a and PtRSH4b contain domains with strong homology to bacterial (p)ppGpp synthetase domains, a glycine residue corresponding to G240 in *Streptococcus equisimilis* Rel (Rel_Seq_) and previously shown to be essential for synthetase activity in bacterial RSH^24,25^ is substituted by an alanine (PtRSH4a) or a serine residue (PtRSH4b). Substitution of this glycine residue in the RSH enzymes of land plants has also been associated with the loss of (p)ppGpp synthetase activity^11,14^. In contrast, PtRSH1 bears well conserved (p)ppGpp hydrolase and synthetase domains. This analysis suggests that PtRSH1 may be a bifunctional enzyme, unlike the monofunctional RSH that have so far been identified in plants and green and red algae.

In addition to the N-terminal catalytic domains, all the three PtRSH also possess a C-terminal regulatory domain (CTD), as observed in bacterial RSH enzymes but with significant modifications (Figs. 1, S2). The PtRSH1 CTD has many of the domains found in the CTD of bacterial RSH including a Threonyl-tRNA synthetase, GTPase, SpoT/RelA (TGS) domain, a helical domain, a zinc finger domain (ZFD) and an Aspartate kinase, Chorismate mutase, TyrA (ACT) domain. PtRSH4a and PtRSH4b have only a partial TGS domain. In *E. coli*, the RelA CTD inhibits (p)ppGpp synthesis, and upon amino acid limitation the CTD binds to the ribosome in association with uncharged tRNA to activate (p)ppGpp synthesis^26^. The TGS domain of RelA interacts with the uncharged tRNA, while the ZFD and ACT domains are involved in binding the ribosome^27–29^. Interestingly, although modelling indicates that the overall structure of the TGS, ZFD and ACT domains is conserved in PtRSH1, we found that the majority of the residues implicated in the binding of RelA to tRNA and the ribosome are not conserved. Furthermore, the ZFD domain lacks the conserved cysteine residues usually found in bacterial RSH (Fig. S3). In the light of these findings it is therefore difficult to determine whether PtRSH1 is able to associate with the ribosome in a similar manner to RelA, and we consider ribosome binding even more unlikely for PtRSH4a and PtRSH4b that possess truncated TGS domains. Another possibility is that the CTD of the *P. tricornutum* RSH are involved in other regulatory interactions. Indeed, in addition to ribosome binding the TGS and ACT domains of other prokaryotic RSH enzymes are implicated in a number of protein-protein and protein- small molecule interactions^30,31^.

We then analysed the expression of the *P. tricornutum RSH* genes in published datasets to determine first whether they are expressed, and second whether *RSH* transcript abundance changes in response to shifts in environmental conditions. *PtRSH1*, *PtRSH4a* and *PtRSH4b* transcripts were identified in a wide-range of RNAseq, microarray and EST experiments^32–42^. All three genes are also expressed under standard growth conditions (Fig. S4,A)(Table S1)^36^. The depletion of nitrogen and other nutrients over long culture times leads to a substantial increase in *PtRSH4a* transcript abundance (Fig. S4,A)^36^. Consistent with a potential role in the acclimation to nutrient deprivation, independent studies show that nitrogen, phosphate and iron deprivation are also accompanied by an increase in *PtRSH4a* transcript abundance (Fig. S4,B–D)(Table S1)^32,33,40,41^. *PtRSH1* showed similar but more modest responses, while *PtRSH4b* showed either no change or a decrease in transcript abundance under these conditions (Fig. S4,D). *PtRSH4b* alone also showed evidence of diurnal regulation with significantly lower transcript abundance in the evening and at the beginning of the night (Fig. S4,E)^34,40,42^. There is also evidence for altered transcript abundance in response to biotic stress such as during copepod grazing where *PtRSH4b* transcript levels significantly decrease^39^ or after treatment with 2E, 4E decadienal, a volatile oxylipin associated with diatom stress signalling that causes an increase in *PtRSH4a* transcript abundance (Table S1)^37,38^. Interestingly, plant *RSH* gene expression is also affected by biotic stress^43–46^.

### Catalytic activities of the *P. tricornutum* RSH enzymes

Next, we investigated the potential catalytic activities of PtRSH1, PtRSH4a and PtRSH4b by expression in *E. coli* strains where the endogenous RSH genes, *relA* and *spoT*, are mutated.

**Figure 2:**
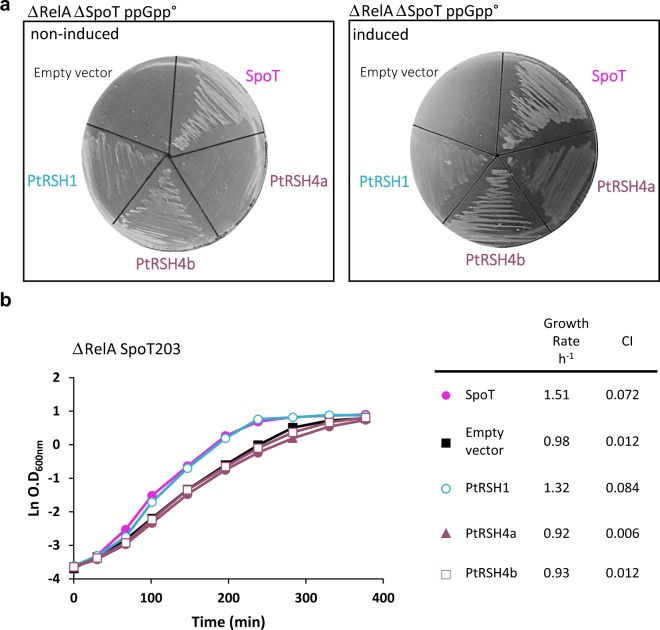
Catalytic activity of *P. tricornutum* RSH. (**A**) The coding sequences for mature PtRSH1, PtRH4a and PtRSH4b were introduced into the pBAD24 plasmid in MG1655 ∆relA ∆spoT. Cells were incubated at 37 °C on M9 minimal agar medium without amino acids in the absence or presence of the inducer arabinose. Empty vector and pBAD24-Spot from *E.coli* were included as controls. Complementation was also observed without induction, presumably due to leaky expression from pBAD24. (**B**) Growth curves and growth rate of MG1655 ∆relA spoT203 containing the coding sequences for the indicated enzymes in the plasmid pBAD24. The values in the growth curve are means ± SD of three biological replicates. CI, 95% confidence interval.

To assay (p)ppGpp synthetase activity, we expressed the *P. tricornutum* RSH enzymes in a ∆*relA* ∆*spoT* mutant. This mutant is unable to grow on minimal medium, and growth can be restored by complementation using a plasmid expressing a functional (p)ppGpp synthetase. The expression of PtRSH1, PtRSH4a and PtRSH4b restored growth to the ∆*relA* ∆*spoT* mutant on minimal medium, as did expression of the positive control *spoT* gene from *E. coli* (Fig. 2A). Thus, all three PtRSH enzymes have (p)ppGpp synthetase activity.

We then used an assay that we recently developed to detect (p)ppGpp hydrolase activity using the ∆*relA spoT*203 mutant strain^11^. In this mutant strain SpoT is defective for (p)ppGpp hydrolase activity while retaining (p)ppGpp synthetase activity, which results in the constitutive over-accumulation of (p)ppGpp and a slow growth phenotype. Normal growth can be restored by expression of an enzyme with (p)ppGpp hydrolase activity such as SpoT. We found that the expression of the diatom PtRSH1 rescued the growth of this mutant in a similar fashion to expression of the positive control *spoT* (Fig. 2B). In contrast, expression of PtRSH4a and PtRSH4b in the ∆*relA spoT*203 mutant strain did not restore normal growth, and we even observed slower growth, perhaps due to enhanced (p)ppGpp biosynthesis. The absence of detectable (p)ppGpp hydrolase activity in PtRSH4a and PtRSH4b is consistent with the divergent (p)ppGpp hydrolase domains in these enzymes that lack many of the residues essential for activity. All together, these results strongly suggest that PtRSH4a and PtRSH4b are exclusive (p)ppGpp synthetase enzymes, and that PtRSH1 is a bifunctional RSH enzyme with both (p)ppGpp synthetase and hydrolase activity.

### *P. tricornutum* RSH enzymes possess a conserved (p)ppGpp synthetase active site

**Figure 3:**
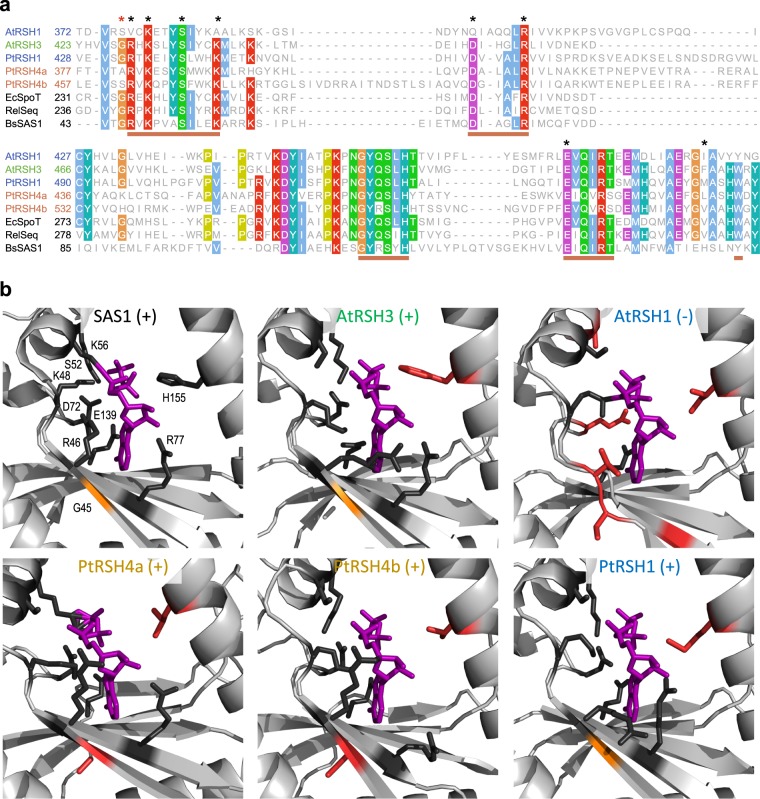
Comparative modelling of the synthetase domain of *P. tricornutum* RSH. (**A**) Amino acid alignments for the (p)ppGpp synthetase domain of the *B. subtilis* SAS1 and *P. tricornutum* (Pt)*, A. thaliana* (At), *E. coli* (Ec), *Streptococcus equisimilis* (RelSeq) RSH. The alignment was performed using MAFFT with default parameters, and coloured with Clustal colouring. Pink horizontal bars indicate important catalytic motifs for (p)ppGpp synthesis (Steinchen & Bange, 2016). (**B**) The structure of the *B. subtilis* SAS1 active site bound to the non-hydrolysable ATP analog AMP-CPP (purple) compared to the corresponding domain in modelled structures of RSH from PtRSH1 (template modelling (TM) score = 0.76 ± 0.10), PtRSH4a (TM score = 0.73 ± 0.11), PtRSH4b (TM score = 0.71 ± 0.11), *A. thaliana* RSH1 (TM score = 0.79 ± 0.09) and *A. thaliana* RSH3 (TM score = 0.88 ± 0.07). The SAS1 conserved glycine at position 45 is shown in orange, and other residues implicated in AMP-CPP binding are shown in black. In the modelled structures conserved AMP-CPP binding residues are indicated in black and non-conserved residues in red. Gly45 and AMP-CPP-binding residues are also indicated by asterisks in the upper panel. (+) denotes evidence of (p)ppGpp synthetase activity, (−) denotes an absence of evidence for (p)ppGpp synthetase activity.

All three RSH from *P. tricornutum* have unusual characteristics. As shown above, PtRSH1 is a bifunctional (p)ppGpp synthetase and hydrolase, a class of enzyme common in bacteria but not yet described in plants or algae. Furthermore, PtRSH4a and PtRSH4b are (p)ppGpp synthetases, despite the lack of a conserved glycine residue previously shown to be critical for (p)ppGpp synthesis as discussed above^24,25^(Fig. 3A). Therefore, to gain functional insights into these characteristics, we modelled the structures of the synthetase domains of the three *P. tricornutum* RSH enzymes using I-TASSER^47^(Fig. 3B). For the three PtRSH enzymes as well as for CRSH and RSH1 from *A. thaliana* we obtained models based on the crystal structure of Small Alarmone Synthase 1 (SAS1) from *Bacillus subtilis* bound to the non-hydrolysable ATP analog α,β-methyleneadenosine 5′-triphosphate (AMP-CPP)^48^. Within the active site of SAS1 the conserved glycine residue G45 (corresponding to Rel_Seq_ G240) is positioned on a beta strand neighbouring R46, a residue involved in binding AMP-CPP in cooperation with a number of other residues (Fig. 3A,B). In PtRSH4a and PtRSH4b the side-chains of the non-conserved residues corresponding to G45 did not greatly affect the active site structure, and their side chains projected away from the active site (Fig. 3B). In both cases, the neighbouring arginine residue corresponding to SAS1 R46 was still predicted to bind AMP-CPP. Interestingly, in the residues corresponding to R46 the arginine side-chain took several different orientations in AtRSH3, PtRSH4a, PtRSH4b and PtRSH1, all of which appear to aid in retaining the adenine and ribose moieties of AMP-CPP.

In AtRSH1, which lacks (p)ppGpp synthetase activity^11^, the residue corresponding to R46 is substituted by a valine, whose side-chain does not retain AMP-CPP and due to its polarity cannot bind with the oxygens in the ribose ring (Fig. 3B). Many other conserved residues involved in AMP-CPP binding are also missing from AtRSH1. In contrast, the synthetase active site of PtRSH1 more closely resembles that of an active (p)ppGpp synthetase with the conservation of most residues involved in AMP-CPP binding. Furthermore, the arginine corresponding to SAS1 G46 takes a similar orientation to the same residue in AtRSH3, which acts as a ppGpp synthetase (Fig. 3B). These modelling results are consistent with the results of our activity assays (Fig. 2), and show that the residue corresponding to SAS1 G45 can be substituted by at least an alanine (PtRSH4a) or a serine (PtRSH4b) residue, without necessarily affecting the conformation of the active site or the orientation of the neighbouring arginine.

### Evolution and domain organization of *P. tricornutum* and other algal RSH enzymes

**Figure 4:**
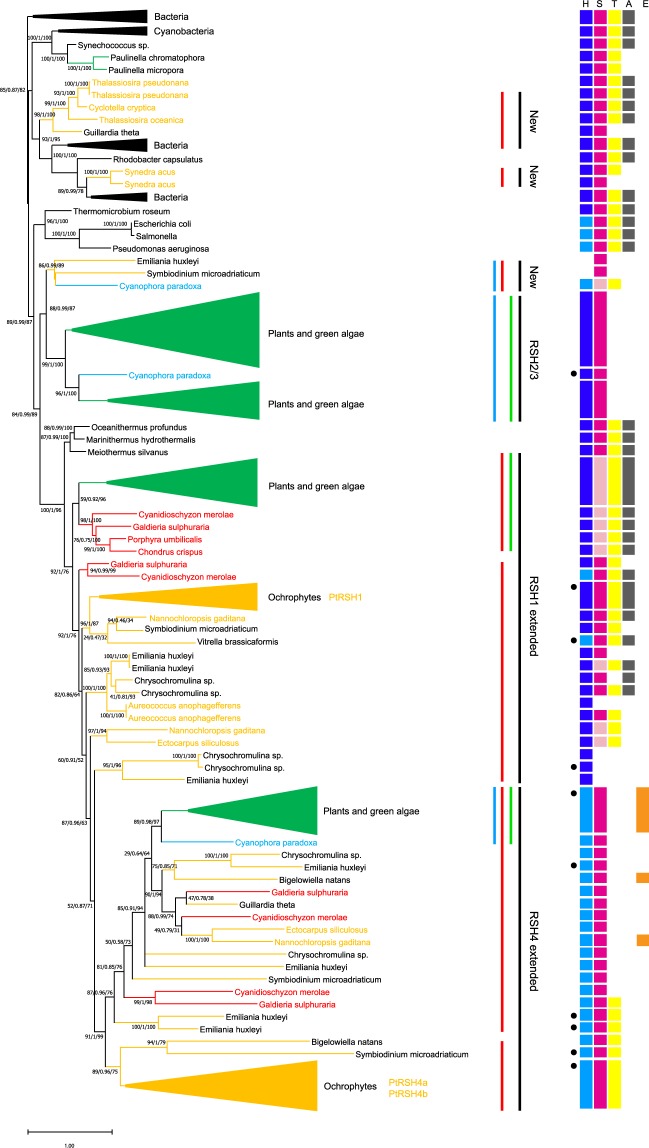
Phylogenetic tree of RSH proteins from diatoms and other photosynthetic organisms. The synthetase and hydrolase domains of different RSH proteins were used to infer phylogenetic relationships using maximum likelihood reconstructions. Diatom RSH are indicated by orange text and branches, CASH RSH are indicated by black text and orange branches, red algal RSH are indicated by red branches, RSH from plants and green algae are condensed and indicated by green branches, glaucophyte RSH are indicated by cyan branches. The scale bar indicates the number of substitutions per sites. Statistical support for branches is shown at the nodes (SH-aLRT/aBayes/Bootstrap). RSH groups are indicated to the right. Domain architecture is shown to the right of the phylogenetic tree: H, (p)ppGpp hydrolase; S, (p)ppGpp synthetase; T, TGS; A, ACT; and E, EF hand. Pale coloured HYD and SYN domains columns indicate a likely loss of activity due to the substitution of important residues. The presence of other domains is indicated by a black circle, and these are listed in Table S2.

Our work shows that the *P. tricornutum* RSH enzymes have significant differences with known plant and algal RSHs. However, the diatom RSH enzymes have not yet been placed within a phylogenetic context. This is necessary for determining whether they belong to new or existing RSH clades, and for understanding when the RSH enzymes were acquired during the complex evolutionary history of the diatom plastid. Therefore, we constructed a phylogenetic tree based on the sequences of the hydrolase and synthetase domains of RSH enzymes from bacteria, representatives of the Archaeplastidae (red algae, glaucophytes, plants and green algae), and algae resulting from secondary or more complex endosymbiosis events (Fig. 4).

We found that PtRSH4a and PtRSH4b belong to a clade that is monophyletic with a sister clade containing the plant and green algae RSH4 enzymes, and which together can be considered as part of an extended RSH4 clade (Fig. 4). The extended RSH4 clade is characterised by the presence of a degraded (p)ppGpp hydrolase domain and a conserved (p)ppGpp synthetase domain (Fig. 4, Table S2). PtRSH4a and PtRSH4b are members of two well separated diatom RSH clades that form part of a larger clade composed of RSH from the ochrophytes and other CASH lineages (organisms descended from an endosymbiosis between a eukaryote and a red alga: Cryptomonads, Alveolates, Stramenopiles and Haptophytes)^49^. We will refer to this clade as the red RSH4 clade, based on the presence of RSH from organisms containing red-lineage plastids and the absence of RSH from the plants and green algae. Within the red RSH4 clade the substitution of the residue equivalent to SAS1 G45 in the (p)ppGpp synthetase active site that we observed in PtRSH4a and PtRSH4b is nearly ubiquitous. The sister plant and algal RSH4 clade does not contain diatom RSH4, but does contain RSH4 from red algae, several CASH lineages and the glaucophyte *C. paradoxa*. This clade can therefore be considered a red and green RSH4 clade based on chloroplast ancestry, although the relationships between RSH from the red algae and CASH lineages appear complex or are not fully resolved. The existence of two monophyletic RSH4 clades in the extended RSH4 clade suggests that the common ancestor of the Archaeplastidae was likely to have possessed two RSH4 like enzymes that were subsequently retained, or selectively lost/duplicated in the different lineages.

PtRSH1 likewise belongs to a red algal and CASH lineage clade (or red RSH1 clade) that is monophyletic with a sister clade containing the plant and green algal RSH1 enzymes (red and green RSH1 clade), and which together can be considered as part of an extended RSH1 clade (Fig. 4). The extended RSH4 clade is characterised by the presence of an intact (p)ppGpp hydrolase domain, as well as by the presence of conserved regulatory TGS and ACT domains in the CTD (Fig. 4, Tables S2). In contrast to the red and green RSH1 clade, most members of the red RSH1 clade possess an intact (p)ppGpp synthetase domain, suggesting that these enzymes have the potential for bifunctionality as we demonstrated for PtRSH1 which is a member of this clade. As observed in previous analyses of plant and algal RSH enzymes we found that the RSH1 enzymes group closely with RSH enzymes from the Deinococcus–Thermus bacterial phylum^12,16,44^, and may also have given rise to the RSH4 clade as suggested by the less well supported CASH groups in the extended RSH1 clade^12^.

We did not detect any diatom RSH in the RSH2/3 clade that has members from the glaucophyte *C. paradoxa* and the plants and green algae. However, we identified a well-supported new CASH and glaucophyte clade in close proximity. This new clade did not share the intact (p)ppGpp hydrolase domain that is characteristic of members of the plant and green algal RSH2/3 clade. Our analysis also revealed that other diatom species possess additional RSH enzymes to those found in *P. tricornutum*. Unlike the *P. tricornutum* RSH, these additional RSH enzymes are not in clades that are monophyletic with RSH enzymes from plants or green algae, and form two new and well-supported clades within the bacterial RSH (top of Fig. 4). These novel RSH clades are unlikely to be sequencing artefacts because there are multiple representatives from different algal species.

### Domain organization of *P. tricornutum* and other algal RSH enzymes

In terms of domain structure, most plant and green algal RSH4 have acquired a C-terminal EF-hand domain (Fig. 4, Table S2). Two CASH RSH4 in the red and green RSH4 clade also possess EF-hand domains, though at the N-terminus. No EF-hand domains could be detected in PtRSH4a and PtRSH4b or indeed any members of the sister RSH4 clade that contains only CASH and red algal RSH. However, a C-terminal MoaD-like protein domain was encountered in three separate members of this clade (Table S2). We also observed new single domain acquisitions in both clades of the extended RSH4 family: a major intrinsic protein domain in the alveolate *Symbiodinium microadriaticum*, a minor capsid protein VI domain in the alveolate *Vitrella brassicaformis*, a phosphopantetheinyl transferase domain in the haptophyte *Emiliania huxleyi*, and a forkhead domain in the green alga *Chlorella variabilis* (Table S2). Interestingly, Ito *et al*. (2017) previously reported the acquisition of tetratricopeptide repeat domains at the C-terminus of RSH4 from some green algae species, acquisitions that were also detected here.

In the extended RSH1 family, the majority of enzymes, including PtRSH1, contain both TGS and ACT domains in the CTD (Figs. 1, S3). This RSH architecture strongly resembles that found in bacterial RSH enzymes, where it is involved in ribosome associations, protein-protein and protein- small molecule interactions^26^. However, there is currently no evidence of such interactions for plant or algal RSH with the exception of a TGS-dependent interaction between Arabidopsis RSH1 and ObgC that was shown in yeast two-hybrid experiments^50,51^. Overall there are only a few examples of domain acquisition within the extended RSH1 family which include an RSH1 from the brown alga *E. siliculosus* that possesses a Chlorophyll A-B binding protein domain, and an RSH1 from the haptophyte *Chrysochromulina* sp that possesses an endomucin domain.

## Discussion

In most bacteria, nutrient limitation or stress provokes the rapid synthesis of the two nucleotide alarmones, pppGpp and ppGpp. In *E. coli*, (p)ppGpp synthesis is controlled by two enzymes, RelA and SpoT. Chloroplast localised RelA SpoT homologue (RSH) enzymes are also found in algae and plants, where they are implicated in the control of chloroplast function and plant development^3,52^. Little is known about RSH enzymes in diatoms, or indeed the entire red algal lineage. In this report, we showed that the genome of the diatom *P. tricornutum* encodes three RSH enzymes: PtRSH1, PtRSH4a and PtRSH4b. Analysis of their amino acids sequences revealed the presence of N-terminal extensions that were predicted to be a chloroplast targeting peptide for PtRSH1 and potentially PtRSH4a. This suggests that at least some of these enzymes function within the chloroplast, the site of (p)ppGpp synthesis and its action in plants^3^. Using heterologous expression in *E. coli* (p)ppGpp mutants we showed that all three *P. tricornutum* RSH are (p)ppGpp synthetases, and that PtRSH1 is also a (p)ppGpp hydrolase (Fig. 2). Analysis of existing gene expression data indicates that the *P. tricornutum RSH* are all expressed under standard growth conditions, and that PtRSH4a in particular is induced in response to nutrient deprivation (Fig. S3, Table S1)^32,33,36,40,41^. Finally, phylogenetic analysis indicated that PtRSH1, PtRSH4a and PtRSH4b are typical members of well-defined diatom RSH clades that can be considered to belong to previously identified plant and algal RSH1 and RSH4 families (Fig. 4)^12,16^. These results indicate that the *P. tricornutum* RSH enzymes and their orthologues are sufficient for the establishment of a (p)ppGpp homeostasis mechanism in diatoms.

PtRSH1 is unusual in that it functions as a bifunctional (p)ppGpp synthetase/hydrolase RSH. Bifunctional RSH such as SpoT are common in bacteria, but no RSH enzymes that are bifunctional like SpoT have been demonstrated in the photosynthetic eukaryotes before now. We note however that members of the plant and green algal RSH2/3 family often possess almost intact hydrolase domains in addition to active synthetase domains (Fig. 4). However, hydrolase activity has not so far been demonstrated for one of these enzymes and their overexpression in plants or bacteria results in (p)ppGpp accumulation^10,11^. Interestingly, our phylogenetic analysis suggests that all members of the red RSH1 clade may be bifunctional (Fig. 4). This is in contrast to the sister red and green RSH1 clade where the majority of RSH1 appear to have lost (p)ppGpp synthetase activity due to the loss of essential residues in the active site^11,16^. Our results are also supported by modelling of the active sites of PtRSH1 and Arabidopsis RSH1 (Fig. 3b). Arabidopsis RSH1, while preserving the global topology of the synthetase active site, has lost the arginine residue with a guanidinium group that is likely to retain ATP in its binding pocket. Structural data indicates that bifunctional RSH can switch between (p)ppGpp-hydrolase-OFF/(p)ppGpp-synthase-ON and hydrolase-ON/synthase-OFF configurations^24^. Our heterologous expression results suggest that PtRSH1 is likely to be capable of undergoing similar activity switches (Fig. 2). The regulation of PtRSH1 and other members of the red RSH1 clade may therefore be significantly different to plant and green algal RSH1 and may reflect the different lifestyles of these organisms.

PtRSH4a and PtRSH4b are also unusual among (p)ppGpp synthetases due to the substitution of a residue corresponding to SAS1 G45/Rel_Seq_ G240. This conserved residue has previously been shown to be necessary for (p)ppGpp synthetase activity^24,25^ and substitution of this residue is widely used to infer the loss of (p)ppGpp synthetase activity^13,14,16,17,53^. Modelling of the active site of PtRSH4a and PtRSH4b shows that the glycine substitution does not appear to affect ATP binding by the neighbouring arginine (Fig. 3b), and sequence analysis indicates that the glycine substitution is widespread in the ochorophyte RSH4 clade. These data suggest that the previous reports of an association between the substitution of the residue corresponding to SAS1 G45 and the loss of synthetase activity may be conditional on the presence of other specific residues in the active site. PtRSH4a and PtRSH4b, and nearly all members of the red RSH4 clade lack the EF-hand domain found at the C-terminal of many members of the sister red and green RSH4 clade. A certain number of novel domain acquisitions were also observed within both the major RSH4 clades in addition to those previously identified in the red and green RSH4 clade^16^. Altogether, these findings suggest that extended RSH4 family enzymes act exclusively as (p)ppGpp synthetases, and are susceptible to domain acquisition, presumably for new regulatory functions. Indeed, altered regulation might be expected, because many enzymes from diatoms involved in processes such as CO_2_ assimilation, sulphate assimilation have different regulatory properties than their orthologues in plants^54,55^. Determining the functions of these domains in (p)ppGpp metabolism presents a fascinating challenge for future research.

We show here that, when the RSH of genome-sequenced diatoms are considered, the evolutionary history of RSH enzymes in the photosynthetic eukaryotes is considerably more complex than previously thought^12,16,44^. We extend the described RSH1 and RSH4 families by showing the existence of sister clades specific to the red-lineage that show distinct functional and structural properties (Figs. 2–4). Interestingly, although Atkinson *et al*. (2011) included several CASH and red algal RSH1 enzymes in their phylogenetic analysis, they did not detect a clear separation of the RSH1 family into a red RSH1 and a red and green RSH1 clade, or the existence of a red RSH4 clade. The difference between these studies is very likely to be due to our use of many more CASH RSH sequences, which are available today thanks to the ever growing list of sequenced CASH genomes. For the same reason we also detected two new CASH RSH families that are not monophyletic with any of the known RSH families in photosynthetic eukaryotes.

Previous reports on the phylogenetic relationships of bacterial, plant and algal RSH enzymes have proposed that plant and algal RSH may have arisen through lateral gene transfer rather than vertical descent from the ancestral chloroplast^12,16,44^. A major reason for this proposition is the grouping of the RSH1 family with RSH from the prokaryotic Deinococcus–Thermus phylum rather than cyanobacteria. Our analysis supports this idea by also showing that the extended RSH1 family groups with RSH from the Deinococcus–Thermus phylum (Fig. 4). Furthermore, our discovery of new CASH RSH families that group with bacteria are evidence that additional lateral gene transfers from bacteria may have occurred recently. This would suggest that lateral transfers of RSH genes can occur readily.

Our report sheds new light on the RSH enzyme family and (p)ppGpp metabolism in the diatoms, and reveals many surprises. Further research is now required to elucidate the mechanism and role of (p)ppGpp signalling in the lifestyle of this important and diverse group of photosynthetic eukaryotes.

## Materials and Methods

### Cloning of *P. tricornutum* RSH

Genomic DNA from *P. tricornutum* Bohlin (strain number CCAP 1052/1A from Culture Collection of Algae and Protozoa, CCAP, Scottish Marine Institute) was isolated from cells grown in late-exponential phase, using an optimized method for diatoms^56,57^. Sequences for the *P. tricornutum* RSH proteins: PtRSH1, PtRSH4a and PtRSH4b (accession codes: 11099, 7629 and 3397, respectively) were retrieved from Joint Genome Initiative (JGI) database (https://genome.jgi.doe.gov/Phatr2/Phatr2.home.html). The amino acid sequences of PtRSH1 and PtRSH4a reported in JGI were partial, so a manual search based on nucleotide sequence was performed to obtain the full-length amino acid sequences. The three PtRSH sequences are given in Fig. S1. The genes coding for the mature proteins without the bipartite chloroplast targeting peptide were amplified by PCR using the primers listed in Table S3. The theoretical transit peptide cleavage site was estimated from^58^ and by using alignments with plant RSH enzymes. Primers were designed to have 20-bp flanking regions homologous to the plasmid (pBAD24) each side of the *NcoI* and *Hind*III restriction sites. The plasmid construction was carried out using the Sequence and Ligation Independent Cloning (SLIC) method^59^. PCR was performed using genomic DNA of *P tricornutum*, the specified primers and Q5 DNA polymerase (New England Biolabs). Since the gene encoding PtRSH1 has an intron, the complete cloned gene was used as a template for the amplification of the two exons using the primers specified in Table S1. The final construct was assembled in the pBAD24 plasmid using SLIC cloning^59^. All constructs were verified by sequencing (GATC Biotech).

### RSH activity assays

To test (p)ppGpp synthetase activity, pBAD24 plasmids containing the *PtRSH* genes, SpoT or without a coding-sequence (empty) were transformed into *E. coli* strain EB425 (MG1655 ∆*relA* ∆*spoT*)^60^ and grown at 37 °C on M9 minimal medium agar plates without amino acids and in the presence or absence of 0.1% arabinose. To test (p)ppGpp hydrolase activity, the same plasmids were transformed into *E. coli* strain EB544 (MG1655 ∆*relA spoT*203)^61^. Pre-cultures from independent colonies for each replicate were diluted in 50 ml Luria Bertani medium containing ampicillin (100 µg mL^−1^) and arabinose (0.1% w/v). Growth was performed at 37 °C under agitation at 175 rpm and optical density was measured at 600 nm every 30 min.

### Prediction of plant and algal RSH enzyme structures

Iterative Threading ASSEmbly Refinement (iTASSER)^47^ was used to predict the structure of the full length Arabidopsis and *P. tricornutum* RSH or the synthetase domain (default settings). The synthetase hydrolase regions were chosen by selection of the regions corresponding most closely to the synthetase region of *Bacillus subtilis* SAS1: AtRSH1, residues 347–542; AtRSH3, residues 404–559; PtRSH1, residues 403–584; PtRSH4a, residues 352–537; PtRSH4b, residues 432–631. The *B. subtilis* SAS1 crystal structure (9decA) was then manually selected in iTASSER for modelling. Results were visualized in PyMOL (Version 2.0 Schrödinger, LLC).

### Phylogenetic analysis of RSH proteins from diatoms and other photosynthetic organisms

Using *P. tricornutum* RSH proteins as queries, a set of homologous proteins was built using public data at the National Center for Biotechnology Information (NCBI) and JGI, as well as individual genome projects: *Synedra acus* (http://www.lin.irk.ru/sacus/index.php?r=site/page&view=downloads&lang=en), *Cyclotella cryptica* (http://genomes.mcdb.ucla.edu/Cyclotella/download.html), *Pseudo nitzschia multistriata* (https://zenodo.org/record/495408#.W1sOJsIyXIU) and *Asterionella formosa* (unpublished data, A. Villain, personal communication). RSH proteins were selected only from fully sequenced genomes, and all predicted RSH per genome were used in the subsequent analysis. Multiple-sequence alignments of homologous proteins were performed separately on both datasets using MAFFT v7.402^62^ with option –auto. Initial alignment was trimmed to include only the hydrolase and synthetase domains by selecting columns 26 to 343 from *E. coli* SpoT. Alignment columns containing gaps in >30% sequences were then removed and phylogenetic reconstructions were created using the web-server^63^ for IQ-TREE version 1.6.11^64^ using default settings, with LG + F + R7 automatically selected as the best fit evolutionary model based on BIC values by ModelFinder^65^. Branch support was tested using three methods: ultrafast boostrap approximation using 1000 bootstraps^66^, the non-parametric Shimodaira–Hasegawa–like approximate likelihood-ratio test (aLRT)^67^, and a Bayesian-like transformation of aLRT (aBayes)^68^. A phylogenetic reconstruction was also created using RAxML^69^ version 8.2.1269 with model PROTGAMMALGX and 100 rapid bootstraps. The topology of the RAxML phylogenetic tree was similar to that produced by IQ-TREE. The alignments and trees are available in Supplementary Dataset 1. Protein domain architecture was analysed by the NCBI CD-search algorithm with default parameters and an E-value threshold of 0.01^70^.

## Supplementary information

Supplementary Figures S1-S4 Tables S1 and S3

Table S2

Dataset 1

## Data Availability

Any datasets generated during and/or analysed during the current study that are not included in the supporting information are available from the corresponding authors on request.
