# Supplementary material for: RSH enzyme diversity for (p)ppGpp metabolism in Phaeodactylum tricornutum and other diatoms
Source: Sci Rep. 2019 Nov 27;9:17682. doi: 10.1038/s41598-019-54207-w (PMC6881373; doi:10.1038/s41598-019-54207-w)
Supplement: Supplementary file 1 — Supplementary Figures S1-S4 Tables S1 and S3 [file 41598_2019_54207_MOESM1_ESM.pdf]

RSH enzyme diversity for (p)ppGpp metabolism in *Phaeodactylum tricornutum* and other diatoms.

L Avilan, C Puppo, A Villain, E Bouveret, B Menand, B Field and B Gontero

Supplementary Data

Fig. S1 Full amino acid sequences of the three RSH proteins found in *P. tricornutum*.
Fig. S2 Alignment of *P. tricornutum* RSH enzymes
Fig. S3 Comparative modelling of the C-terminal domain of *P. tricornutum* and Arabidopsis RSH.
Fig. S4 Alterations in *P. tricornutum RSH* transcript abundance in response to environmental perturbation.
Table S1 Number of expressed sequence tags representing the RSH genes per 10,000 total EST in *P. tricornutum* EST databases.
Table S3 List of the primers used in this study.

References

Fig. S1 Full amino acid sequences of the three RSH proteins found in *P. tricornutum*.

>PtRSH1-11099
MQVSSTGGMWTILLLAFLWSCTCIAFTTPRWFPTPNMRHSQSIVPLFDRIPPSFPSLEMAGANTTGSGMNSAQTLFP
TPDRTSLASIYTSESHTAAAAVMSNGFNYSAHYNTAALIPPVTFTSASTEVAAEKDRSEADTPPEVNHWTEIMRVGW
KDDTSTTNSSTTSAQISSSSTKWDAVWSDYDLQQIERYWDRIMPTVSYLGTDAVAKIYQALCVAYRAHRGQMRKSGE
PFIVHPVEVSLLLSGLKMDAETVMSGLLHDTVEDTDLTFQQVETLFGHTVRSIVEGETKVSKLPKLAFAEYADEQAE
NLRQMFVAMTSDYRIIIVKLADRLHNMRTLRYMKSEKQIKISRETLDIFAPLAHRMGIWQFKSELEDTSFMYLYPQE
YKRLNRRLRLHQQSFQETLEKAQDILQRQLNLDSTLQQQAYKVEVSGRTKEIYSLWHKMETKNVQNLDHIVDVVALR
VVISPRDKSVEADKSELSNDSDRGVWLCYHALGLVQHLPGFVPVPTRVKDYISFPKPNGYQSLHTALILNGQTIEVQ
IRTSMMHQVAEYGMASHWAYTDDKRRGSNEELYSTPWLSSIKEWQNEAVSSRDFVDSVRRELLGKRVFVFLRNGKIL
NLARGATAIDAAFQIHTEVGLSMHGVEINGKPVPLSYELQNGDVVSILTGSGRPATDWMRYAKSRSTRSKLRSYFRD
RQKESLREAGKILLMDYLTVHGTLIQESSYLEKDFAIPKSTEELEYLLPGKTSFNDVDELLVGIGKNHNRSKLHQIV
SQLFEVPKRILITAEEKKIPRLPSNIFAAVQLRQDRAKDAGDAVDLVGEIVTDPATWSTLPKKSVFSYSAESMLAGL
DLPIEYADPEHLCVDCLPVYEDEIVGTRKSGSVSIPMVHRVGCPHAQRAINQAKAHQRQKPFVSKLQNVTSVPGTSI
PRPQLRVDSVSLRQTYGKTAPWMRRAGGQTSKYKSGTVDLPVKLQWSDLDEKDSLFLSEIVVHCGDRKLLLADCSEV
VSETVEIIKTGSSTNEETATLVFLVRVGGLGHIQTLMDRLMKVRSVLSVERRFGSELR

>PtRSH4a-7629
MTNGIPAATATAAAATPTLPLSTHRRRRRMPTVLSTAALLLSSRCSQSADAFTALHNGFRSSKTSLAFRSNVMDGLR
TDALLSTSSSSPTSTIGPLPTWLSYPQAHLKDSLVAELSQAMKVSFFTETETLQLLAAVEEAAGGDAHKVAGTADFL
RILVETMEMGLNALVAAAFHYADCVELREHTRLQSSTSQTAAAMVRHANLDAYYGEHVSQIADDAGRLKQLEWVAQV
VMQTHASRASPDAHDAENLRQLLLSETRDWRALAIRAGACLYRLRGLLKSDSYELTPERVRVGREALSIYAPLASRL
GMHRLKNELEGAAFRVLYQRQYQAVNAMAKEVQTKDENNNMRDVLAEVKNDLTLLLQKDPEFSKAVSDFTVTARVKE
SYSMWKKMLRHGYKHLLQVPDALALRIVLNAKKETPNEPVEVTRARERALCYYAQKLCTSQFAPVANAPRFKDYVER
PKPNGYQSLHYTATYESWKVEIQVRSGEMHQVAEFGLASHWDYKASQDSLAEADVEPSDLDQSSDAYVRKVQEWHWD
QHNGVAPATVEWDASPASFAPVTASDIWQSRIRAERIRARTQRLEPYLQALTAAQSDLAREYVFCFLKSGDTPKVLA
LPAGACVLDALRQGGVDGSVQLNGVEASITRQLTNGDVLTISSLAVV

>PtRSH4b-33947
MKSGLLDYTTLTVGISLLHKRNGNTMKAKKRPRAERIQVGKPSASAFSWWSLFLIEWSSLPVFGLTQWSLYGNEFTT
QSKPTELRAIQSPVRNDAVSPRKKKEILLDFPDSPSSRTGEHVNSEFSPQNRIGLPPCCLPYDHFTAEEIEVEVGWL
QYSLLDHGVSFDDVRQIVSTIYNVSENNTSVTVGIVQFLRLLFDTCGEEAHMDRMLSTSVVLASVYHYAECMEAHNQ
GSTAYLLGNANLRNKEMPHNRASIDSEGTVLPAIRGEDTLTRDIISPKPMPRRLRSSTGSFGAGDEVFLITEGAARI
KRAEALVQSVIGNGHIISQAESDLFRDWLLSVMDDWRSLAIRVFACLYRLEGIRLDAGTYDGRTPEVVKLAKEAMRV
YSPLAGRLGMYRLKSRLDEEAFRILYRRQYNAVSSLYLESGAAMEAVSNILRTKISVALQQDESLMMQLEGLEVSSR
VKQPYSFWKKLLKKRTGGLSIVDRRAITNDSTLSIAQVQDGIALRVIIQARKWTENEPLEEIRARERFFCYYVQHQI
RMKWPEVEADRVKDYILYPKPNGYRSLHHTSSVNCNGVDFPFEVQVRSDEMHMIAEYGVAAHWGYKLGNILAPSSPS
VGACAGMLPPAKQCCEILSPAFRPGFSFSHTRLGSTQSFADALVDAKETLLEQYVYVFISGISDESDGQLLSLPAKS
LVVDALAAMDKIDASNLRVLLNGKRVKLDDVVENGDVLMVVA

Fig. S2 Alignment of *P. tricornutum* RSH enzymes. Amino acid alignments for RSH from *P. tricornutum*, *A. thaliana* (At), *E. coli* (Ec), *S. equisimilis* (RelSeq) and the Small Alarmone Synthase (SAS) 1 from *B. subtilis*. The alignment was performed using MAFFT with default parameters. Blue and pink horizontal bars indicate important catalytic motifs for (p)ppGpp hydrolysis and synthesis (Steinchen & Bange, 2016).

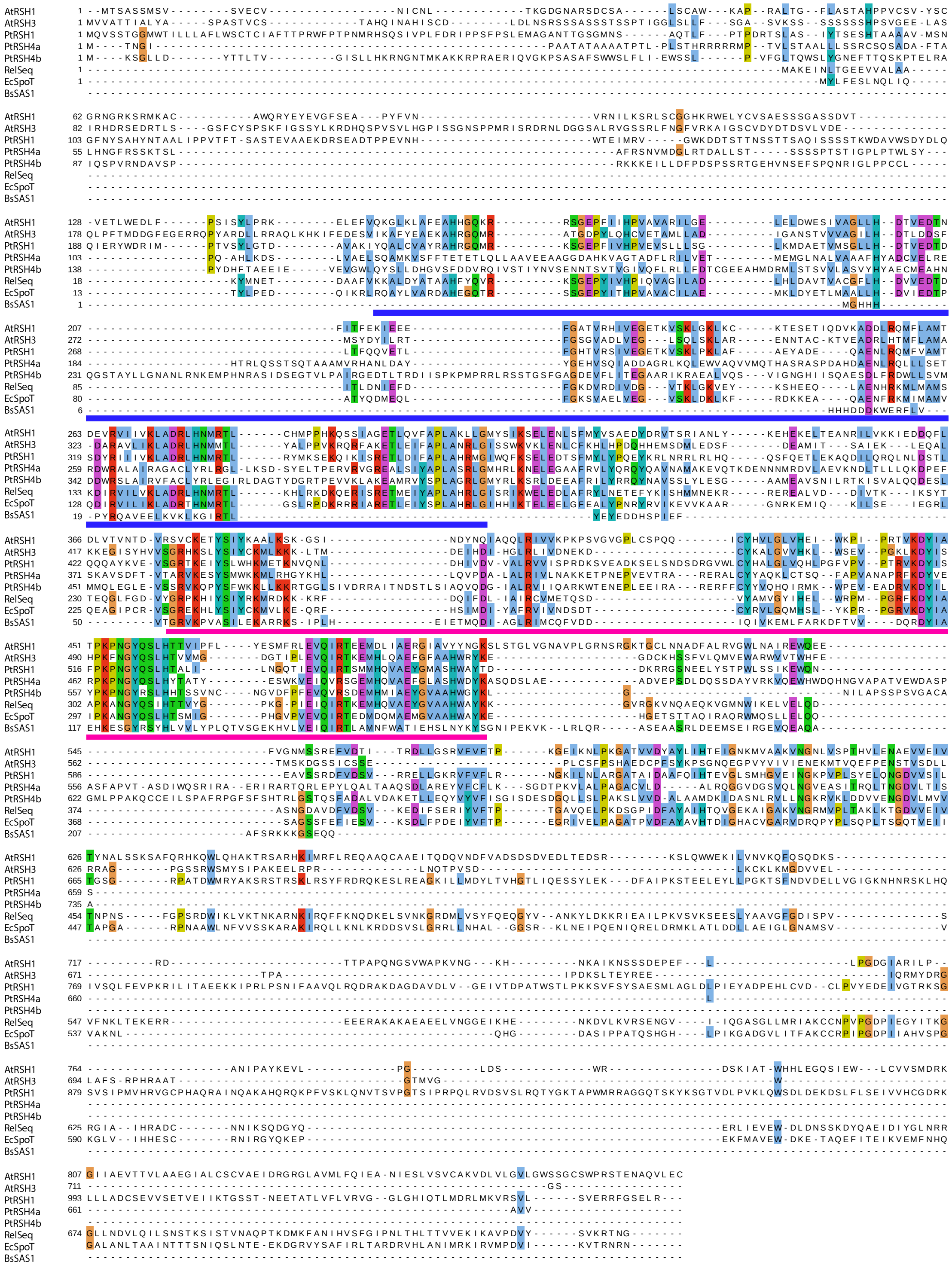

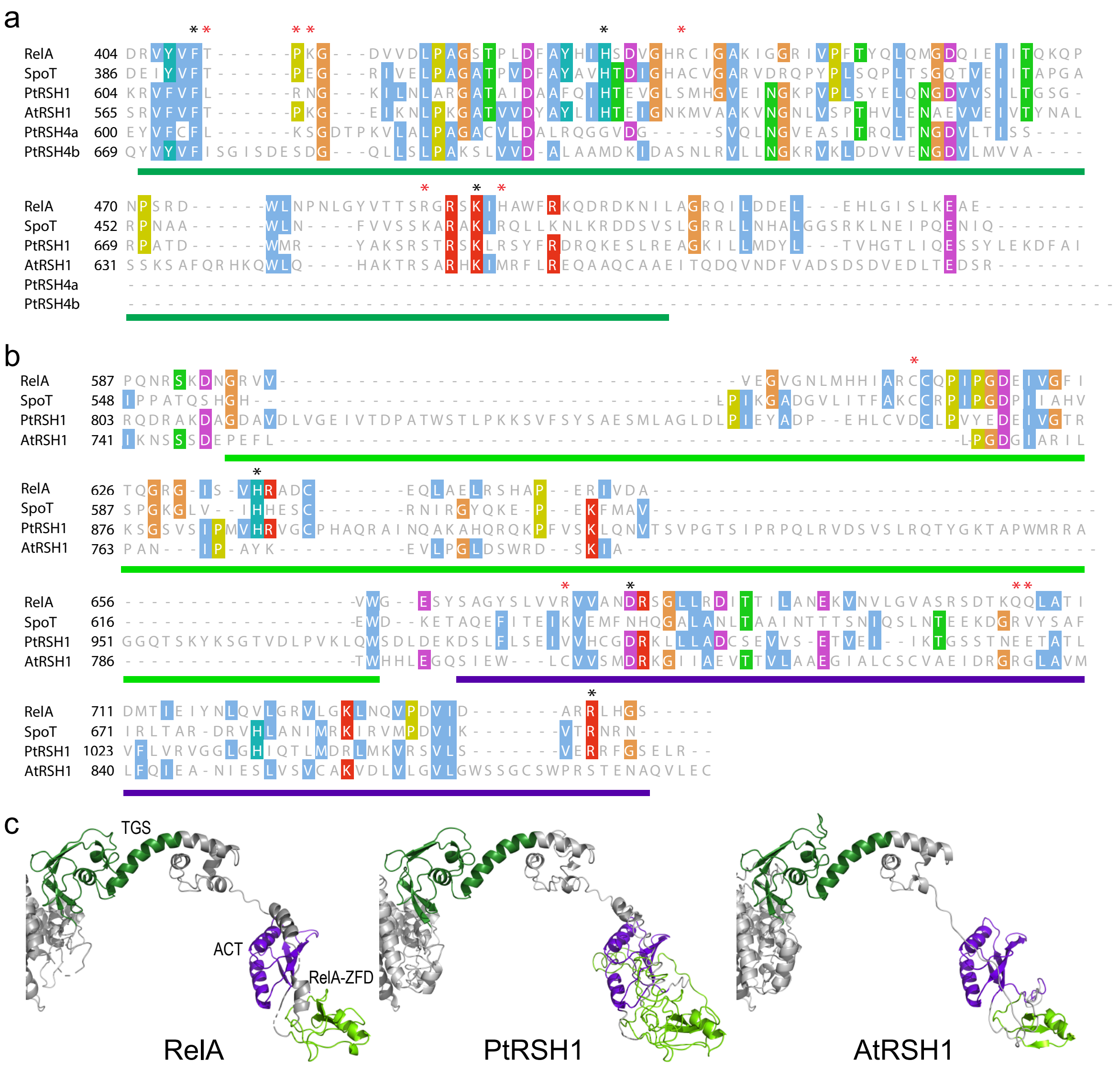

Figure S3 Comparative modelling of the C-terminal domain of *P. tricornutum* and Arabidopsis RSH. Amino acid alignments of regions of the regulatory C-terminal domain of the *E. coli* SpoT and RelA, *P. tricornutum* (Pt) RSH1, and *A. thaliana* (At) RSH1 containing **(A)** the TGS domain (green horizontal bars) and **(B)** the RelA ZFD domain (light green bars) and the ACT domain (purple bars). Residues implicated in binding the tRNA in the TGS domain and the ribosome in the ZFD and ACT domains are indicated by asterisks, black indicates that the residue is conserved between RelA and PtRSH1 and orange indicates that the residue is not conserved. The alignment was performed using the MAFFT algorithm with default parameters, and is coloured with Clustal colouring. **(C)** The structure of the ribosome bound *E. coli* RelA C-terminal domain (PDB 5KPV, Loveland *et al.,* 2016) compared to the corresponding domain in ITASSER modelled structures of PtRSH and AtRSH1.

Fig. S4 Alterations in *P. tricornutum RSH* transcript abundance in response to environmental perturbation. RNAseq and microarray experiments were analysed to specifically determine how *RSH* transcript levels change in response to different treatments. (a) *RSH* expression following transfer of NH_4 grown cells to N-free media for 2 h, and transfer to media containing NO_3 with sampling over 228 h. The mean Fragments Per Kilobase of transcript per Million (FPKM) of three biological replicates +/- SE is shown. Data from McCarthy et al. (2017). (b) *RSH* expression following transfer of cells from nitrogen replete media to nitrogen free media (-N). The average log2 fold change for treated cells versus non-treated cells is presented for three biological replicates. Differences are significant unless marked by ns, for not significant. Data from Matthijs et al. (2016). (c) Average log2 *RSH* fold change for phosphorous deprived cells versus non-treated cells for three biological replicates. Data from Alipanah et al. (2018). (d) Average log2 *RSH* fold change for treated cells versus non-treated cells. Data from a diurnal timecourse of *P. tricornutum* in the presence of different concentrations of Fe, Smith et al. (2016).

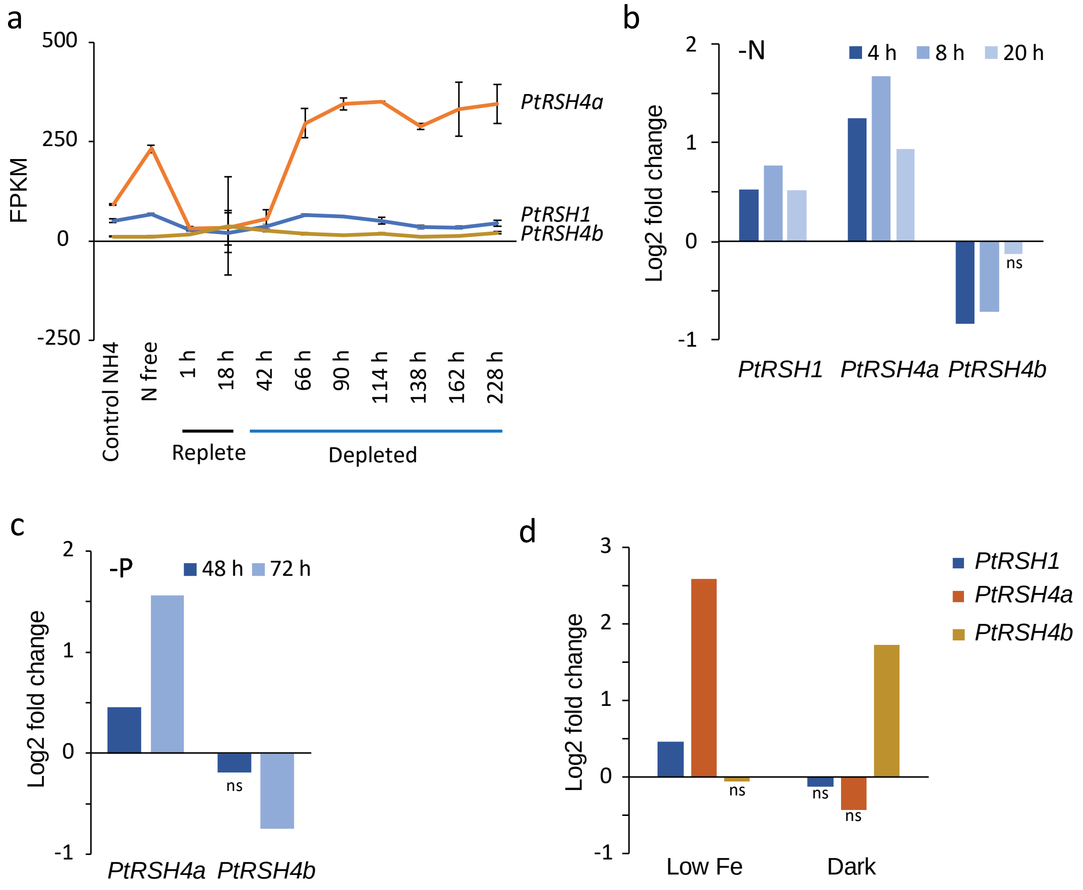

Table S2 List of the primers used in this study.

Name	Sequence (5’ -3’)	Description
11099 fw exon1<br>11099 rev exon2	GCAGGAGGAATTCACCATGGCAATCTACACCTCGGAATCCCA<br>CCGCCAAAACAGCCAAGCTTCTAGCGCAACTCCGAACCAA	Cloning of the PtRSH1(complete gene)
11099 rv exon1<br>11099 fw exon2	CTTGCCATACCGTATTCAGCTACTTGATGC<br>GCTGAATACGGTATGGCAAGTCACTGGGCG	SLIC for the spliced PtRSH1 gene.
7629 fw<br>7629 rev	AGCAGGAGGAATTCACCATGGACGGTCTCCGTACCGATGC<br>CCGCCAAAACAGCCAAGCTTTCAAACCACCGCGAGGGACG	Cloning of the PtRSH4a
33947 fw<br>33947 rev	CAGGAGGAATTCACCATGGGTCCAGACTCTCCAAGCAGTC<br>CCGCCAAAACAGCCAAGCTTTTATGCTACAACCATCAAAACATC	Cloning of the PtRSH4b

References

Alipanah L, Winge P, Rohloff J, Najafi J, Brembu T, Bones AM. 2018. Molecular adaptations to phosphorus deprivation and comparison with nitrogen deprivation responses in the diatom Phaeodactylum tricornutum. PLoS One. 2018 23:13:e0193335

Levitan O, Dinamarca J, Zelzion E, Gorbunov MY, Falkowski PG. 2015. An RNA interference knock-down of nitrate reductase enhances lipid biosynthesis in the diatom Phaeodactylum tricornutum. Plant J. 84:963-73.

Loveland AB, Bah E, Madireddy R, Zhang Y, Brilot AF, Grigorieff N, Korostelev AA. 2016. Ribosome•RelA structures reveal the mechanism of stringent response activation. Elife 5, e17029.

Matthijs M, Fabris M, Broos S, Vyverman W, Goossens A. 2016. Profiling of the Early Nitrogen Stress Response in the Diatom Phaeodactylum tricornutum Reveals a Novel Family of RING-Domain Transcription Factors. Plant Physiol. 170:489-98.

McCarthy JK, Smith SR, McCrow JP, Tan M, Zheng H, Beeri K, Roth R, Lichtle C, Goodenough U, Bowler CP, Dupont CL, Allen AE. 2017. Nitrate Reductase Knockout Uncouples Nitrate Transport from Nitrate Assimilation and Drives Repartitioning of Carbon Flux in a Model Pennate Diatom. Plant Cell. 29:2047-2070

Smith SR, Gillard JT, Kustka AB, McCrow JP, Badger JH, Zheng H, New AM, Dupont CL, Obata T, Fernie AR, Allen AE. 2016. Transcriptional Orchestration of the Global Cellular Response of a Model Pennate Diatom to Diel Light Cycling under Iron Limitation. PLoS Genet. 12: e1006490.

Steinchen W, Bange G. 2016. The magic dance of the alarmones (p)ppGpp. Mol Microbiol 101, 531-544.
