# Supplementary figures and images for: RSH enzyme diversity for (p)ppGpp metabolism in Phaeodactylum tricornutum and other diatoms
Source: Sci Rep. 2019 Nov 27;9:17682. doi: 10.1038/s41598-019-54207-w (PMC6881373; doi:10.1038/s41598-019-54207-w)

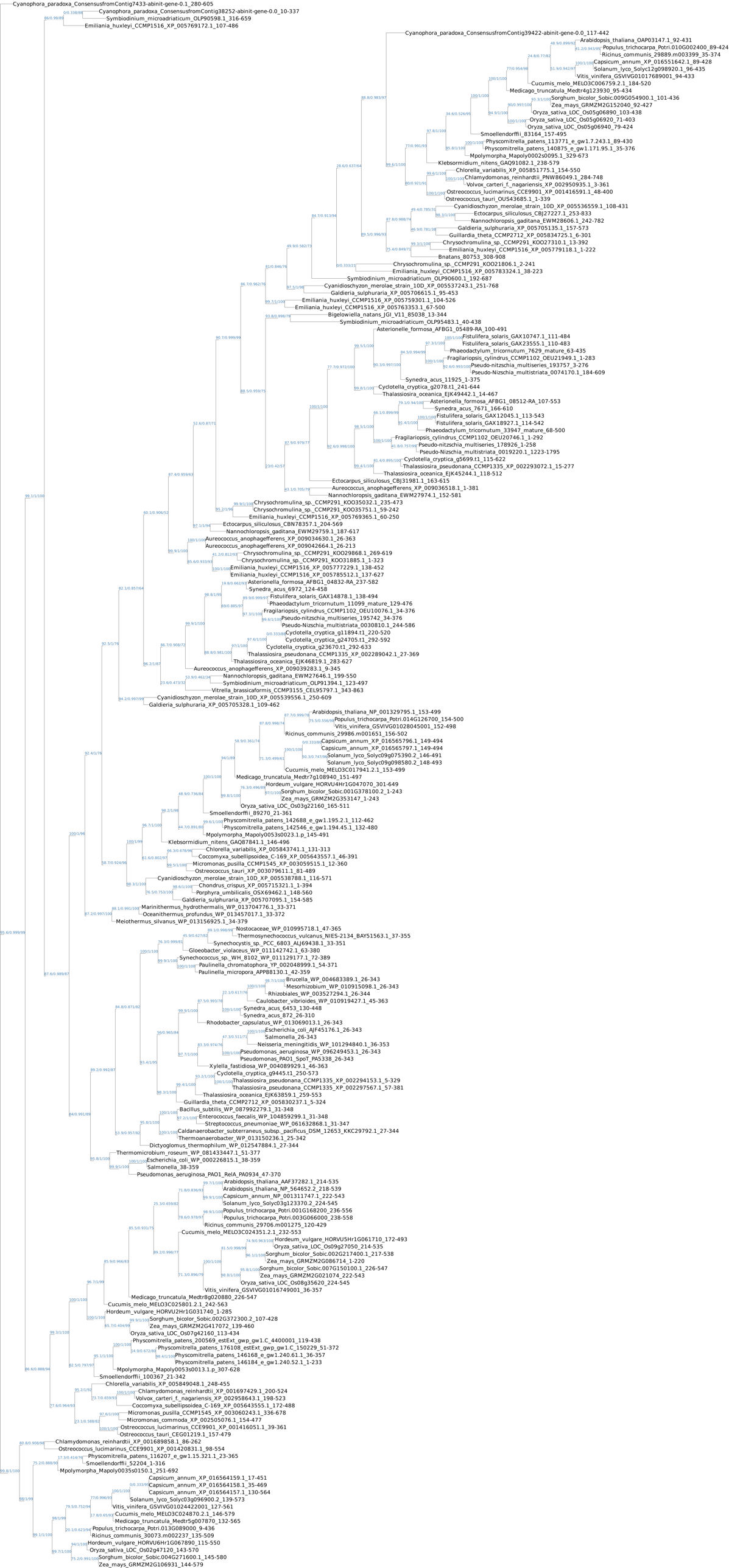

Supplement: Supplementary file 3 — Dataset 1 [file 41598_2019_54207_MOESM3_ESM.zip › Supplementary Dataset 1/IQTREE/RSH_hd_syn_all_final_aligned_26-343ecoli-SpoT_cleaned30.fa.treefile.pdf]
